# Occult pleural dissemination detected intraoperatively in patients with thymic tumors: a retrospective analysis

**DOI:** 10.1186/s13019-021-01717-2

**Published:** 2021-11-18

**Authors:** Zuodong Song, Shu Zhu, Tangbing Chen, Weigang Zhao

**Affiliations:** 1 Shanghai Lung Cancer Center, Shanghai Chest Hospital, Shanghai Jiao Tong University, Shanghai, 200030 China; 2 Department of Ultrasound, Shanghai Jiao Tong University Affiliated Sixth People’s Hospital, Shanghai, 200233 China; 3 Department of Thoracic Surgery, Shanghai Chest Hospital, Shanghai Jiao Tong University, Shanghai, 200030 China; 4 Department of Thoracic Surgery, Shanghai Jiao Tong University Affiliated Sixth People’s Hospital, Shanghai, 200233 China

**Keywords:** Occult pleural dissemination, Thymic tumor, Intraoperatively

## Abstract

**Background:**

Thymic tumors usually present with adjacent organ invasion or pleural dissemination, but very few studies have reported on occult pleural dissemination detected intraoperatively. This study aimed to investigate the risk factors that can predict pleural dissemination preoperatively.

**Methods:**

Consecutive patients with thymic tumors who underwent surgery from January 2010 to January 2017 were reviewed. Only patients without pleural dissemination detected preoperatively were included in this study. Demographic, clinical, pathological, and survival data were collected for statistical analysis. Further analyses were performed to find the risk factors of occult pleural dissemination.

**Results:**

A total of 352 patients with thymic tumors were included in this study. Seven patients had pleural dissemination detected intraoperatively. All pleural dissemination cases were in clinical Masaoka-Koga stage III, and most underwent the video-assisted thoracoscopic surgery (VATS) approach (or VATS exploration). Univariate analysis showed that positive squamous cell carcinoma (SCC) antigen was the only predictor of pleural dissemination (*p* = 0.009). Tiny nodules close to the diaphragm were detected in the computed tomography scans of 1 case after reviewing the imaging data. Tumor recurrence occurred in 5 patients during follow-up. The disease-free survival rates were better in patients with a solitary nodule than those with multiple nodules (*p* = 0.019). No significant difference was detected in terms of disease-free survival rates between SCC antigen positive and SCC antigen negative patients.

**Conclusions:**

Positive SCC antigen was the only detected risk factor for predicting pleural dissemination in thymic tumors preoperatively in this study. The VATS approach (including VATS exploration) is suggested for patients with clinical Masaoka-Koga stage III and SCC antigen positive thymic tumors, according to our experience.

## Introduction

Thymic epithelial tumors (TETs) are rare malignancies that usually occur in the anterior mediastinum in adults. Thymic tumors are usually indolent [1], and complete surgical resection is considered necessary to achieve long-term patient survival [2]. However, recurrence rates in patients who have undergone surgical resection remain high [3–10]; the pleura is the most common site of thymic tumor recurrence after radical resection [11–13]. Though several studies on post-surgery-occurring pleural implants have been conducted, only one group reported occult pleural dissemination that was detected intraoperatively; however, their sample size was small [14]. As more accurate preoperative staging would likely lead to improved treatment planning, particularly in terms of the optimal surgical approach, we aimed to identify risk factors for occult pleural dissemination in patients with TETs.

## Methods

### Study design and patients

Data of consecutive patients with thymic tumors who had undergone surgery between January 2010 and January 2017 were extracted from a prospectively maintained database; those with preoperative pleural dissemination were excluded from the study. As only anonymized data were used, the requirement for informed consent was waived by our hospital’s institutional review board.

Patient records were reviewed for demographic and clinicopathological characteristics, treatment details, and survival results. Demographic information included age and gender. Clinicopathological characteristics included comorbidity, serum tumor marker, perioperative treatments, clinical stage, surgical approach and WHO histological type. Univariate analysis was performed to identify the risk factors of occult pleural dissemination.

### Follow-up and recurrence

Based on our department’s follow-up protocol, all patients underwent cervical and abdominal ultrasonography, chest computed tomography (CT), and serum tumor marker evaluation every 3 months during the first 2 years post-surgery and every 6 months thereafter. Demographic, clinical, pathological, and survival data were collected for statistical analyses.

Disease-free survival (DFS) was calculated from the date of surgery to that of tumor recurrence or death. Recurrence was defined as suggested or confirmed reemergence of lesions in the resected tumor bed, anterior mediastinum, adjacent tissues, pleura, pericardium, diaphragm, or distant organs. Only confirmed recurrence events as evidenced through radiological followed by pathological examination were documented.

### Statistical analysis

Continuous variables were compared using Student’s t-test and are expressed as means and standard deviations. Categorical variables were compared using Pearson’s χ^2^ test or Fisher’s exact test. Associations between occult pleural dissemination and clinical values were determined using univariate binary logistic regression analysis. Kaplan–Meier curves and log-rank tests were used to estimate survival probabilities and to compare DFS between groups. Two-sided *P*-values < 0.05 indicated statistical significance. All statistical analyses were performed using SPSS (version 23.0; IBM Corp., Armonk, NY, USA).

## Results

A total of 352 patients with TETs were included in this study; their clinical and pathological characteristics are shown in Table 1. Two hundred patients (56.8%) were men, and the average patient age was 52.9 ± 13.2 years. Thirty-three patients (9.4%) had myasthenia gravis at presentation, and 33 (9.4%) received neoadjuvant therapy. Pleural dissemination was detected intraoperatively in seven patients (1.9%); no significant differences were detected between patients with versus without occult pleural dissemination in terms of demographic and clinical characteristics such as age, sex, presence of myasthenia gravis, surgical approach, and histological type. Higher rates of squamous cell carcinoma (SCC) antigen positivity were detected in patients with than in those without occult pleural dissemination (*P* = 0.031). All incidents of occult pleural dissemination were observed in patients with clinical Masaoka–Koga stage III disease, and video-assisted thoracoscopic surgery (VATS) or VATS exploration was performed in most cases (85.7%).

**Table 1:** Demographic and clinicopathological characteristics of the 352 patients with thymic tumors

Characteristics	Patients with PD	Patients without PD	*P*
Age	54.0 ± 8.8	52.9 ± 13.3	0.74
*Gender*			0.687
Male	5	195	
Female	2	150	
*Myasthenia gravis*			0.133 (fisher)
Yes	2	31	
No	5	314	
*CEA*			N/A
Positive	0	3	
Negative	7	312	
*SCC antigen*			0.031 (fisher)
Positive	2	13	
Negative	5	332	
*CYFRA21-1*			N/A
Positive	0	5	
Negative	7	340	
*CA125*			N/A
Positive	0	5	
Negative	7	340	
*NSE*			N/A
Positive	0	2	
Negative	7	343	
*Neoadjuvant therapy*			N/A
Yes	0	33	
No	7	312	
*Clinical stage*			N/A
Masaoka I/II	0	217	
Masaoka III	7	126	
*Surgical approach*			0.702
Sternotomy/thoracotomy	1	97	
VATS	6	248	
*WHO histological type*			0.244
A/AB/B1	1	148	
B2/B3	4	114	
Carcinoma	2	83	

PD, pleural dissemination; CEA, carcinoembryonic antigen; CA125, carbohydrate antigen 125; CA199, carbohydrate antigen 199; NSE, neuron specific enolase; CYFRA21-1, cytokeratin 19 fragment 21–1; SCC, squamous cell carcinoma; N/A, not applicable

**Fig. 1:**
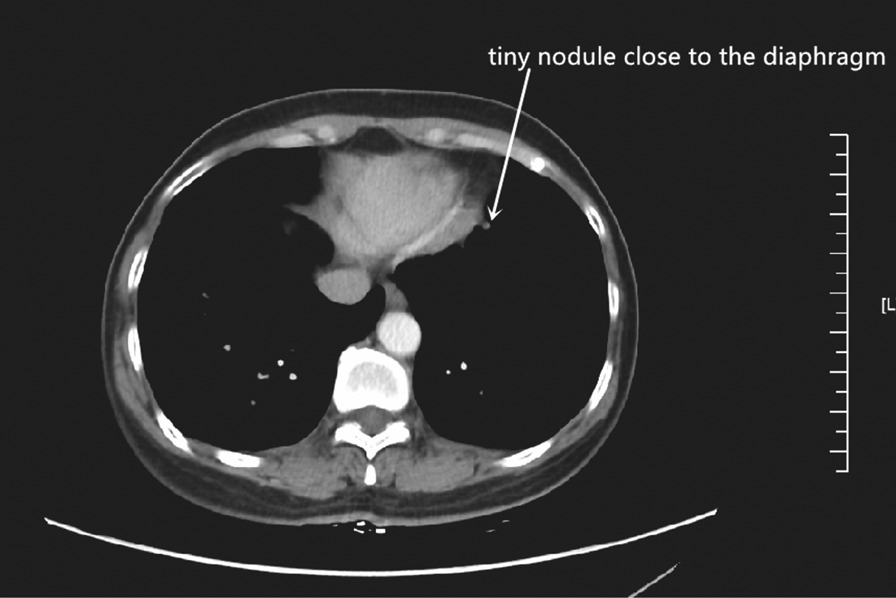
The mediastinal window on chest CT showed a tiny nodule close to the diaphragm

Univariate analysis showed that SCC antigen positivity was the only predictor of occult pleural dissemination in patients with thymic tumors (odds ratio 10.185, 95% confidence interval, 1.804–57.50; *P* = 0.009) (Table 2). Careful review of CT images revealed a tiny nodule close to the diaphragm in one of the patients (Fig. 1).

**Table 2:** Univariate analysis of occult pleural dissemination

Variable	OR	95% CI	*P* value	Coefficient
*Gender*				
Male	Reference			
Female	0.517	0.099–2.703	0.435	−0.659
Age	0.997	0.941–1.055	0.906	−0.003
*Myasthenia gravis*				
No	Reference			
Yes	4.039	0.752–21.688	0.104	1.396
*SCC*				
Negative	Reference			
Positive	10.185	1.804–57.501	0.009	2.321
*Surgical approach*				
Open	Reference			
VATS	2.323	0.276–19.546	0.438	0.843
*WHO histological type*				
A/AB/B1	Reference			
B2/B3	5.158	0.569–46.779	0.145	1.641
Carcinoma	4.452	0.316–39.656	0.305	1.265

WHO, world health organization

All patients with occult pleural disseminations underwent thymectomy plus pleurectomy during surgery, and one patient also received additional hyperthermic intrathoracic chemotherapy (HITOC) during the procedure. Moreover, four patients with multiple pleural nodules received adjuvant treatment after surgery, while three with solitary nodules only required post-surgical observation (Table 3).

**Table 3:** Perioperative and follow-up results of patients with occult pleural dissemination

Case No.	Operation approach	Post-operative treatment	Recurrence	Recurrence date (months)	Number of nodules
1	Thymectomy + pleurectomy	Chemo + RT	YES	13	2
2	Thymectomy + pleurectomy	No	No	69	1
3	Thymectomy + pleurectomy	Chemo	Yes	18	4
4	Thymectomy + pleurectomy	No	No	49	1
5	Thymectomy + pleurectomy	RT	Yes	16	2
6	Thymectomy + pleurectomy	No	Yes	31	1
7	Thymectomy + pleurectomy + HITOC	Chemo	Yes	9	2

HITOC, hyperthermic intrathoracic chemotherapy

**Fig. 2:**
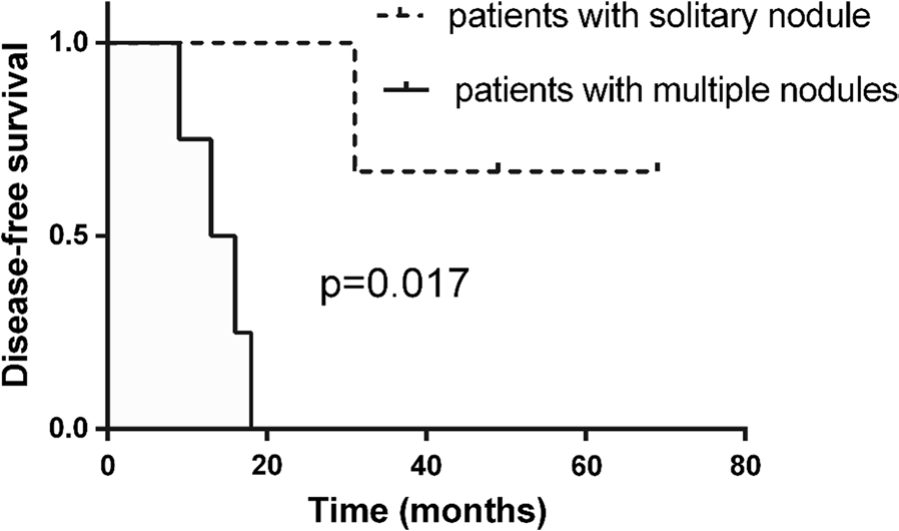
Kaplan–Meier curves for disease-free survival of patients with a solitary pleural nodule and multiple pleural nodules in patients with occult pleural dissemination

**Fig. 3:**
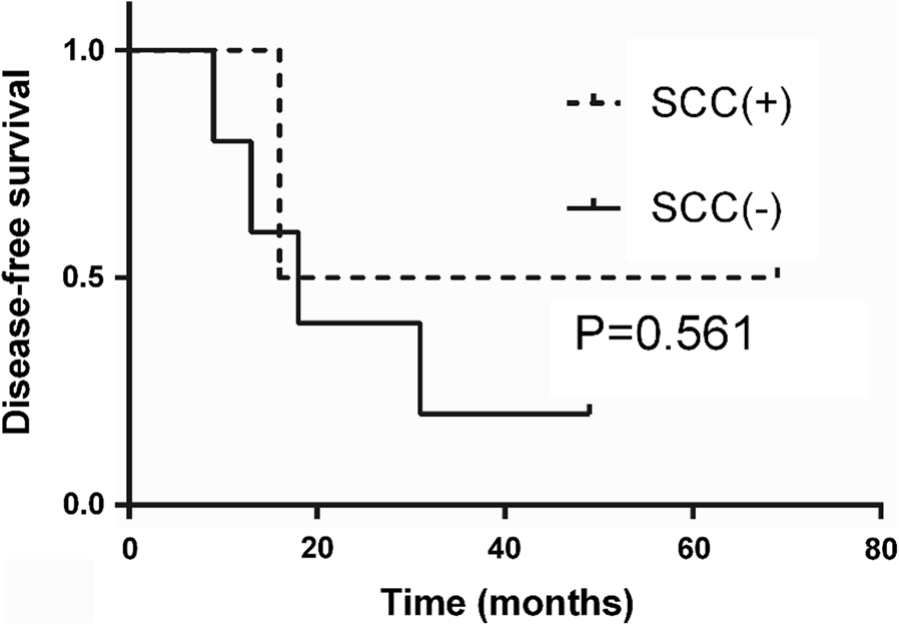
Kaplan–Meier curves for disease-free survival of patients with positive squamous cell carcinoma (SCC) antigen and negative SCC antigen in patients with occult pleural dissemination

At a median follow-up of 18 months (range 9–69 months), 5 patients (71.4%) with occult pleural dissemination developed pleural recurrence after surgery. Patients with a solitary pleural nodule had a more favorable DFS than those with multiple nodules (*P* = 0.017, Fig. 2). No significant difference in DFS was detected between patients with SCC antigen positivity versus negativity in the occult pleural dissemination group (*P* = 0.561, Fig. 3).

## Discussion

Many investigators have explored the management of pleural dissemination in patients with thymic tumors. However, only the study by Kamel et al. investigated the risk factors for pleural dissemination (including those that are occult) [14]. Our study showed that all incidents of occult pleural dissemination were observed in patients with clinical Masaoka–Koga stage III disease, and that most had undergone VATS (or VATS exploration). We also found that SCC antigen positivity was the only predictor of occult pleural dissemination.

Kamel et al. demonstrated that performing a core/surgical biopsy increases the risk of pleural metastasis [14]; such biopsies are likely to disrupt the tumor capsule, causing pleural space contamination. Consequently, preoperative biopsies were not performed for patients with resectable mediastinal tumors at our center; as such, the association between core/surgical biopsies and the occult pleural dissemination of TETs could not be investigated in our study.

Kamel et al. [14] also found that macroscopic capsular invasion was associated with pleural metastasis other than organ invasion, which is inconsistent with our results. A major reason for this discrepancy may be differences in patient characteristics between these two studies; our investigation only included individuals with occult pleural dissemination, whereas Kamel et al.’s included those with all types of pleural dissemination [14]. Notably, we found no significant association between the type of invaded organ and rate of occult pleural dissemination.

The histological classification as devised by the World Health Organization (WHO) is considered an important predictor of thymic tumor recurrence. However, our study showed no independent association between the WHO histological classification and occult pleural dissemination, which is consistent with data from Kamel et al.’s study [14]. We found that B2/B3 thymoma and thymic cancer had higher pleural dissemination rates than A/AB/B1 thymoma (3.4 vs. 0.6% and 2.3% vs. 0.6%, respectively), though these differences were not significant. Our findings may be attributed to the low incidence of occult pleural dissemination of thymic tumors; as such, a significant relationship between the WHO histological classification and occult pleural dissemination rate may be discovered if a larger sample size is examined.

We found that the expression of the SCC antigen, which is generally associated with the presence of SCC, correlated with occult pleural dissemination in patients with TETs. However, the relationship between SCC antigen positivity and thymic SCC has not been studied previously owing to the low incidence of the latter. Our study showed that the histological subtypes of the two SCC antigen-positive patients were not thymic SCCs, suggesting that this antigen may not necessarily be associated with this type of malignancy. Though our study showed that SCC antigen positivity was the only risk factor predictive of occult pleural dissemination in patients with thymic tumors, these results should be interpreted with caution owing to the very low proportion of patients with SCC antigen positivity.

Most patients with occult pleural dissemination in our study underwent VATS or VATS exploration (85.7%). Thymectomy via a median sternotomy is the standard surgical approach for thymic tumors; with advancements in surgical technologies and instrumentation, minimally invasive thymectomy (MIT, which has several advantages over open thymectomy) has become available [15–18]. However, most patients in earlier studies that investigated MIT had early disease stages, and thus, the suitability of MIT for advanced thymic tumor stages remains unclear. All patients in our study with clinical Masaoka–Koga stage III thymic tumors who underwent MIT recovered well; however, the advantages of MIT could not be ascertained because of the lack of a control group. Though VATS was performed more frequently in patients with occult pleural dissemination than in those without (85.7% vs. 71.8%), this difference was not significant. Per our experience, VATS can enlarge the field of vision and help explore concealed locations, making it easier to detect small occult nodules. Therefore, we would still recommend VATS (or VATS exploration) for patients with clinical Masaoka–Koga stage III thymic tumors and/or SCC antigen positivity.

Thymectomy with pleurectomy is generally performed for patients with thymic tumors who have pleural metastasis, thereby prolonging DFS [19]. All patients with occult pleural dissemination in our study underwent thymectomy plus pleurectomy; though they all exhibited encouraging DFS rates, we cannot necessarily attribute these outcomes to the surgical procedures alone because of our small sample size and lack of a control group.

HITOC is currently the main treatment for malignant pleural mesothelioma and has been demonstrated to prolong overall survival [20, 21]. A previous study showed that surgical cytoreduction with HITOC was feasible in select patients and was associated with satisfactory survival rates [22]. Only one patient in the occult pleural dissemination group received additional HITOC but experienced pleural recurrence 9 months after surgery. Whether HITOC is beneficial for pleural dissemination remains unclear, and clinical trials with larger sample sizes are warranted to determine its advantages and disadvantages.

All patients with multiple pleural nodules received adjuvant therapy after surgery, and all those with solitary pleural nodules were subsequently followed upon. Though aggressive multimodality therapy is considered an appropriate treatment for pleural dissemination [19], patients in our study who underwent surgery alone had better DFS than those who were administered multimodality therapy. Reportedly, patients with a small number of disseminated pleural nodules have better prognoses than those with a larger number [23]; therefore, the improved DFS in the surgery-only group could be attributable to the small number of disseminated pleural nodules rather than to the treatment itself.

Considering that SCC antigen positivity was the only predictor of pleural dissemination in patients with thymic tumors, we compared DFS rates between patients who were and were not positive for SCC antigens but found no significant difference; one possible explanation may be the small sample size, while another may be that SCC can only help predict occult pleural dissemination but not DFS. Therefore, additional studies using larger patient populations will be required to determine the relationship between SCC antigen positivity and DFS.

One limitation of this study was that the number of patients with occult pleural dissemination was relatively small, which resulted in severely difference between patients with and without occult pleural dissemination group. However, it is difficult to accumulate more subjects over a short period of time for purposes of increasing the statistical power because of the low incidence rate of occult pleural dissemination in thymic tumors. Another limitation was that the follow-up period was short.

## Conclusions

This study showed that SCC antigen positivity was the only risk factor for preoperative pleural dissemination in patients with thymic tumors. Therefore, it is important to carefully examine CT images acquired before surgery, particularly those depicting the vicinity of the diaphragm. Furthermore, our experience suggests that the VATS approach (including VATS exploration) ought to be recommended for patients with clinical Masaoka–Koga stage III and SCC antigen-positive thymic tumors.

## Data Availability

The data that support the findings of this study are available from Shanghai Chest Hospital and are available from the corresponding author on reasonable request.

## Abbreviations

CT: Computed tomography
DFS: Disease-free survival
HITOC: Hyperthermic intrathoracic chemotherapy
MIT: Minimally invasive thymectomy
SCC: Squamous cell carcinoma
TET: Thymic epithelial tumors
VATS: Video-assisted thoracoscopic surgery
WHO: World Health Organization

## References

[1] Safieddine N, Liu G, Cuningham K (2014). Prognostic factors for cure, recurrence and long-term survival after surgical resection of thymoma. J Thorac Oncol.

[2] Huang J, Detterbeck FC, Wang Z, Loehrer PJ (2010). Standard outcome measures for thymic malignancies. J Thorac Oncol.

[3] Huang J, Rizk NP, Travis WD (2009). Comparison of patterns of relapse in thymic carcinoma and thymoma. J Thorac Cardiovasc Surg.

[4] Blumberg D, Port JL, Weksler B (1995). Thymoma: a multivariate analysis of factors predicting survival. Ann Thorac Surg.

[5] Wright CD, Wain JC, Wong DR (2005). Predictors of recurrence in thymic tumors: importance of invasion, World Health Organization histology, and size. J Thorac Cardiovasc Surg.

[6] Masaoka A, Monden Y, Nakahara K, Tanioka T (1981). Follow-up study of thymomas with special reference to their clinical stages. Cancer.

[7] Monden Y, Nakahara K, Iioka S (1985). Recurrence of thymoma: clinicopathological features, therapy, and prognosis. Ann Thorac Surg.

[8] Regnard JF, Magdeleinat P, Dromer C (1996). Prognostic factors and long-term results after thymoma resection: a series of 307 patients. J Thorac Cardiovasc Surg.

[9] Margaritora S, Cesario A, Cusumano G (2011). Single-centre 40-year results of redo operation for recurrent thymomas. Eur J Cardiothorac Surg.

[10] Okumura M, Ohta M, Tateyama H (2002). The World Health Organization histologic classification system reflects the oncologic behavior of thymoma: a clinical study of 273 patients. Cancer.

[11] Zhang HX, Xiao ZF, Liang J (2015). Patterns and predictors of recurrence after radical resection of thymoma. Radiother Oncol.

[12] Yuan ZY, Gao SG, Mu JW (2017). Long-term outcomes of 307 patients after complete thymoma resection. Chin J Cancer.

[13] Khandelwal A, Sholl LM, Araki T, Ramaiya NH, Hatabu H, Nishino M (2016). Patterns of metastasis and recurrence in thymic epithelial tumours: longitudinal imaging review in correlation with histological subtypes. Clin Radiol.

[14] Kamel MK, Stiles BM, Ghaly G (2016). Predictors of pleural implants in patients with thymic tumors. Ann Thorac Surg.

[15] Kang CH, Hwang Y, Lee HJ, Park IK, Kim YT (2016). Robotic Thymectomy in anterior mediastinal mass: propensity score matching study with transsternal thymectomy. Ann Thorac Surg.

[16] Cheng YJ, Kao EL, Chou SH (2005). Videothoracoscopic resection of stage II thymoma: prospective comparison of the results between thoracoscopy and open methods. Chest.

[17] Gu Z, Chen C, Wang Y (2018). Members of the Chinese alliance for research in thymomas. Video-assisted thoracoscopic surgery versus open surgery for Stage I thymic epithelial tumours: a propensity score-matched study. Eur J Cardiothorac Surg.

[18] Jurado J, Javidfar J, Newmark A (2012). Minimally invasive thymectomy and open thymectomy: outcome analysis of 263 patients. Ann Thorac Surg.

[19] Choe G, Ghanie A, Riely G (2020). Long-term, disease-specific outcomes of thymic malignancies presenting with de novo pleural metastasis. J Thorac Cardiovasc Surg.

[20] Ried M, Potzger T, Braune N (2013). Cytoreductive surgery and hyperthermic intrathoracic chemotherapy perfusion for malignant pleural tumours: perioperative management and clinical experience. Eur J Cardiothorac Surg.

[21] Sugarbaker DJ, Gill RR, Yeap BY (2013). Hyperthermic intraoperative pleural cisplatin chemotherapy extends interval to recurrence and survival among low-risk patients with malignant pleural mesothelioma undergoing surgical macroscopic complete resection. J Thorac Cardiovasc Surg.

[22] Markowiak T, Neu R, Ansari MKA (2021). Surgical cytoreduction and HITOC for thymic malignancies with pleural dissemination. Thorac Cardiovasc Surg.

[23] Okuda K, Yano M, Yoshino I (2014). Thymoma patients with pleural dissemination: nationwide retrospective study of 136 cases in Japan. Ann Thorac Surg.

